# Chromosome-scale reference genome of broccoli (*Brassica oleracea* var. *italica* Plenck) provides insights into glucosinolate biosynthesis

**DOI:** 10.1093/hr/uhae063

**Published:** 2024-02-28

**Authors:** Qiuyun Wu, Shuxiang Mao, Huiping Huang, Juan Liu, Xuan Chen, Linghui Hou, Yuxiao Tian, Jiahui Zhang, Junwei Wang, Yunsheng Wang, Ke Huang

**Affiliations:** College of Horticulture, Hunan Agricultural University, Changsha, Hunan, 410128, China; Engineering Research Center for Horticultural Crop Germplasm Creation and New Variety Breeding, Ministry of Education, Changsha, Hunan, 410128, China; Key Laboratory for Vegetable Biology of Hunan Province, Changsha, Hunan, 410128, China; College of Horticulture, Hunan Agricultural University, Changsha, Hunan, 410128, China; Engineering Research Center for Horticultural Crop Germplasm Creation and New Variety Breeding, Ministry of Education, Changsha, Hunan, 410128, China; Key Laboratory for Vegetable Biology of Hunan Province, Changsha, Hunan, 410128, China; College of Horticulture, Hunan Agricultural University, Changsha, Hunan, 410128, China; Engineering Research Center for Horticultural Crop Germplasm Creation and New Variety Breeding, Ministry of Education, Changsha, Hunan, 410128, China; Key Laboratory for Vegetable Biology of Hunan Province, Changsha, Hunan, 410128, China; College of Horticulture, Hunan Agricultural University, Changsha, Hunan, 410128, China; Engineering Research Center for Horticultural Crop Germplasm Creation and New Variety Breeding, Ministry of Education, Changsha, Hunan, 410128, China; Key Laboratory for Vegetable Biology of Hunan Province, Changsha, Hunan, 410128, China; College of Horticulture, Hunan Agricultural University, Changsha, Hunan, 410128, China; Engineering Research Center for Horticultural Crop Germplasm Creation and New Variety Breeding, Ministry of Education, Changsha, Hunan, 410128, China; Key Laboratory for Vegetable Biology of Hunan Province, Changsha, Hunan, 410128, China; College of Horticulture, Hunan Agricultural University, Changsha, Hunan, 410128, China; Engineering Research Center for Horticultural Crop Germplasm Creation and New Variety Breeding, Ministry of Education, Changsha, Hunan, 410128, China; Key Laboratory for Vegetable Biology of Hunan Province, Changsha, Hunan, 410128, China; College of Horticulture, Hunan Agricultural University, Changsha, Hunan, 410128, China; Engineering Research Center for Horticultural Crop Germplasm Creation and New Variety Breeding, Ministry of Education, Changsha, Hunan, 410128, China; Key Laboratory for Vegetable Biology of Hunan Province, Changsha, Hunan, 410128, China; Hunan Provincial Key Laboratory for Biology and Control of Plant Disease and Insect Pests, Hunan Agricultural University, Changsha, Hunan, 410128, China; College of Horticulture, Hunan Agricultural University, Changsha, Hunan, 410128, China; Engineering Research Center for Horticultural Crop Germplasm Creation and New Variety Breeding, Ministry of Education, Changsha, Hunan, 410128, China; Key Laboratory for Vegetable Biology of Hunan Province, Changsha, Hunan, 410128, China; Hunan Provincial Key Laboratory for Biology and Control of Plant Disease and Insect Pests, Hunan Agricultural University, Changsha, Hunan, 410128, China; College of Horticulture, Hunan Agricultural University, Changsha, Hunan, 410128, China; Engineering Research Center for Horticultural Crop Germplasm Creation and New Variety Breeding, Ministry of Education, Changsha, Hunan, 410128, China; Key Laboratory for Vegetable Biology of Hunan Province, Changsha, Hunan, 410128, China

## Abstract

Broccoli (*Brassica oleracea* var. *italica* Plenck) is an important vegetable crop, as it is rich in health-beneficial glucosinolates (GSLs). However, the genetic basis of the GSL diversity in Brassicaceae remains unclear. Here we report a chromosome-level genome assembly of broccoli generated using PacBio HiFi reads and Hi-C technology. The final genome assembly is 613.79 Mb in size, with a contig N50 of 14.70 Mb. The GSL profile and content analysis of different *B. oleracea* varieties, combined with a phylogenetic tree analysis, sequence alignment, and the construction of a 3D model of the methylthioalkylmalate synthase 1 (MAM1) protein, revealed that the gene copy number and amino acid sequence variation both contributed to the diversity of GSL biosynthesis in *B. oleracea*. The overexpression of *BoMAM1* (BolI0108790) in broccoli resulted in high accumulation and a high ratio of C4-GSLs, demonstrating that BoMAM1 is the key enzyme in C4-GSL biosynthesis. These results provide valuable insights for future genetic studies and nutritive component applications of *Brassica* crops.

## Introduction

The Brassicaceae family generates high-value oilseed, vegetable, and condiment crops via their seed and vegetative tissues [1]. In 2022, ~142.25 million tons of *Brassica* vegetables was produced worldwide, valued at USD 21.19 billion (http://faostat.fao.org/). Based on U’s triangle [2], the basic genomes (A, B, and C) of the *Brassica* genus form three diploid species, *Brassica rapa* (AA, *n* = 10), *Brassica nigra* (BB, *n* = 8), and *Brassica oleracea* (CC, *n* = 9), and can also combine to form three amphidiploids, *Brassica juncea* (AABB, *n* = 18), *Brassica carinata* (BBCC, *n* = 17), and *Brassica napus* (AACC, *n* = 19) [3, 4]. A recent genome duplication divergence event resulting in *Arabidopsis* lineages and the Brassiceae tribe produced several rounds of genomic rearrangement and rediploidization after whole-genome triplication (WGT), leading to the genomes of the current three diploid species [5, 6]. These evolutionary features have created an interesting system involving gene family expansion and multiple gene copies for the study of trait diversity in the Brassiceae tribe.

Multiple *B. oleracea* cultivar groups have been domesticated through artificial selection; this species is integral to human diets in the form of broccoli (var. *italica*), cabbage (var. *capitata*), kale (var. *acephala*), cauliflower (var. *botrytis*), Brussels sprouts (var. *gemmifera*), and kohlrabi (var. *gongylodes*) [7, 8]. Seven *B. oleracea* genomes have been assembled to date [1, 8–12]. In the early investigation of genomic sequencing, a kale (TO1000DH3) genome was highly fragmented due to low assembly coverage, at most 80% of the estimated genome size, in which short reads were used alone [1, 13]. Recently, five *B. oleracea* genotype reference genomes (Korso_1401, OX-heart_923, D134, C-8, and Cap02-12) were improved using PacBio sequencing aided by Hi-C chromosome conformation capture to anchor scaffolds to chromosomes [8–11]. One genotype was sequenced using a combination of Oxford Nanopore Technology and optical maps, demonstrating the utility of these technologies for complex duplicated genomes [12]. Complete genome assembly contributes to the identification of new features of transposable elements (TEs), particularly for long terminal repeat retrotransposons (LTR-RTs), which are an important component of plant genomes, allowing wide variation of content among different species. This technique is beneficial for genetic and genomic comparative studies of *B. oleracea*, and enables the high-quality sequencing of *Brassica* varieties with high nutritional value for molecular breeding and functional genomics investigations.

Broccoli (*Brassica oleracea* var. *italica* Plenck) is rich in anticarcinogenic, antioxidant, and health-promoting components, including polyphenolic compounds, vitamin C, and glucosinolates (GSLs) [14–17]. GSLs are secondary metabolites of broccoli that offer beneficial properties related to human health. Sulforaphane (4-methylsulfinylbutyl isothiocyanate, SF), the hydrolysis product of glucoraphanin (RAA), which is an aliphatic glucosinolate [18, 19], induces phase 2 enzyme activity that converts carcinogens into inactive metabolites [20], thereby reducing the risk of various cancers [21, 22]. At least 120 known GSL structures have been identified in Chinese kale, cabbage, broccoli, and Chinese cabbage [17, 23, 24]. Previous studies based on quantitative trait loci (QTL) mapping, homologous gene alignment, and gene function identification have revealed many genes related to GSL biosynthesis in *Arabidopsis thaliana* and other *Brassica* crops [15, 23, 25, 26]. The species genotype, presence or absence of orthologous genes, derivation of paralogous genes, duplication of genes, tandem repeat of gene families, and tissue-specific expression of genes all contribute to the diversity of GSLs in *Brassica* crops. At present, the reference genome sequences and transcriptomic information provide a solid foundation for improving molecular breeding studies of GSL in *Brassica* crops.

Aliphatic GSLs, derived from Met, have a wider distribution in plants than aromatic or indolic GSLs. The biosynthetic pathway involves three major phases: side chain elongation, core structure formation, and side chain modification [23]. A set of enzymes involved in side chain elongation and modification contribute to the GSL content and structure diversity [27]. The methylthioalkylmalate synthase (*MAM*) gene cluster is a genetic locus central to GSL side chain length, and is related to an insect-resistant quantitative trait locus [28, 29]. The *MAM* cluster comprises three genes (*MAMa*, *MAMb*, and *MAMc*) derived from whole-genome duplication and specialization of *MAM* duplicates [29, 30]. *MAM1* and *MAM2* originate from *MAMa* gene duplication; *MAM-L* (*MAM3*) is orthologous to *MAMb*, and *MAMc* has been lost in evolution [29]. MAM-L provides precursors for aliphatic GSLs with long side chains, whereas MAM2 and MAM1 control short-chain GSLs [29, 31–33]. QTLs encompass three tandem *MAM* genes which were detected in two *B. rapa* BC2DH populations. Naturally occurring insertion within the exon of *BrMAM-3* has resulted in loss of function and low GSL content [34]. The *BrMAM1-A* of the wild mustard A genome is overexpressed in the *Arabidopsis mam1* knockout line background; specifically, the C4-aliphatic GSL content level is elevated and comparable to that of the wild type (WT) [27]. From an evolutionary perspective, MAMs are related to isopropylmalate synthase (IPMS), which catalyzes the first reaction of leucine biosynthesis. IPMS and MAMs belong to the DRE-TIM metallolyase superfamily [35, 36]. The major domains of this protein can assume a closely packed form in the homodimer, in which the N-terminal domain is the catalytic domain, consisting mainly of a (β/α) 8 barrel (TIM barrel) with a divalent metal cofactor necessary for substrate binding. The C-terminal domain contains an allosteric Leu binding site and acts as a regulatory domain [35, 36].

In this study, the chromosomal-level genome of the advanced-generation inbred broccoli line BOP04-28-6 was assembled. Phylogenetic and comparative genomic analyses were performed to resolve the phylogenetic position, WGT event, and chromosome structure of broccoli. As RAA is highly enriched in broccoli, broccoli genome assembly can be used to reveal molecular mechanisms related to RAA-specific accumulation. Novel insights into the hereditary basis of GSL profile diversity and the key genes in C4-aliphatic GSL biosynthesis were obtained through genomic analysis, characterization of GSL biosynthesis genes, and metabolite data generated from different *B. oleracea* varieties. This chromosome-level genome provides an important resource for research on the molecular mechanisms of agricultural traits.

## Results

### Genome sequencing, assembly, and annotation

**Figure 1:**
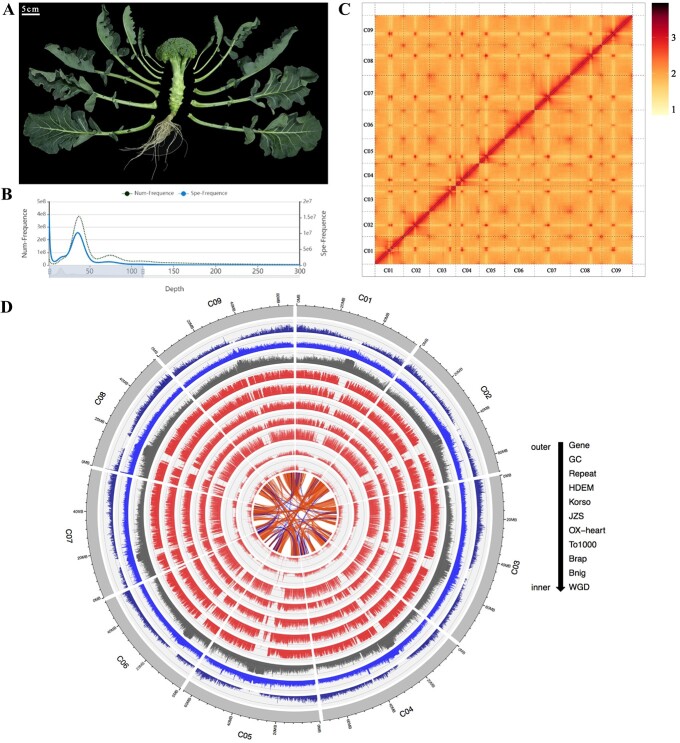
Genome of *B. oleracea* var. *italica* Plenck (Bop04-28-6, broccoli). **A** Image showing key features of a mature period broccoli. **B** 17-mer spectrum of Illumina reads. **C** Hi-C interaction heat map of the assembled Bop04-28-6 genome. Color bar at the right represents the density of Hi-C interactions, which is indicated by the number of links at 1-Mb resolution. **D** Circos display of Bop04-28-6 and other species genomic features.

An advanced-generation inbred line of broccoli that is rich in RAA was used for genomic sequencing (Fig. 1A). We sequenced and assembled this genome by using a hybrid approach that included Illumina sequencing, PacBio SMRT, and a Hi-C chromatin interaction map. Illumina HiSeq sequencing and survey analysis provided an estimated genome size of broccoli of ~655.33 Mb, using GenomeScope with the default parameters, except for a *k*-mer size of 17 (Fig. 1B), with a high homozygosity of 99.56% (Supplementary Data Table S1) and proportion of repeated sequences of 63.23%. This generated 35 Gb of Illumina short reads (53.44-fold coverage) and 29 Gb of PacBio long reads (44.27-fold coverage; Supplementary Data Table S1). The genome size of the final assembly was 613.79 Mb, with 552 contigs (N50 of 14.70 Mb; Table 1). Finally, the chromosome-scale scaffolds were assembled based on Hi-C data (68.37 Gb), and a 576.18-Mb contig was assembled into nine chromosome-scale pseudomolecules (93.87% of the estimated genome size; Figs. 1C and D; Supplementary Data Table S1). In the assessment of the broccoli genome assembly using Core Eukaryotic Gene Mapping Approach (CEGMA) and Benchmarking Universal Single-Copy Orthologs (BUSCO) software to reveal genome completeness, it was found that 99.19% of core eukaryotic genes and 99.4% of the complete single-copy orthologs were intact (Supplementary Data Table S1), indicating high quality of the assembled genome.

The protein-coding gene (PCG) content of the broccoli genome was annotated using an integrated approach consisting of EVidenceModeler, homology, and *de novo* gene prediction, and RNA sequencing (RNA-seq) (Fig. 1D; Supplementary Data Table S1). A total of 55 958 PCGs were predicted (Supplementary Data Table S1), and 97.60% of the predicted PCGs were annotated against multiple protein-related databases [e.g. Swiss-Prot, Gene Ontology (GO), and Kyoto Encyclopedia of Genes and Genomes (KEGG); Supplementary Data Table S1; Supplementary Data Fig. S1]. In addition, 18 467 non-coding RNAs were identified, including 12 061 ribosomal RNAs, 3228 transfer RNAs, 774 microRNAs, and 2404 small nuclear RNAs (Supplementary Data Table S1).

We also predicted and annotated the repeated sequences in the broccoli genome. A total of 361.25 Mb (58.85%) of the assembly was identified as repeat sequences (Supplementary Data Table S1). Most of these were LTR-RTs, which constituted 48.35% of this genome, followed by DNA TEs (5.78%), long interspersed nuclear elements (2.78%), and short interspersed nuclear elements (0.03%). Moreover, 3.87% of repeated sequences could not be classified (Supplementary Data Table S1). Interestingly, LTR-RTs constituted 13% of the broccoli genome, and Gypsy elements accounted for 11.3% (Supplementary Data Table S1). This composition was unlike those of other *Brassica* plants, such as *B. rapa* ‘Chiifu’ [37] (11.3% Gypsy elements, 4.9% Copia elements) and *B. nigra* [38] (20.9% Gypsy elements, 10.4% Copia elements; Supplementary Data Fig. S2A–C).

### Comparative genomic and phylogenomic analyses of Cruciferae

**Figure 2:**
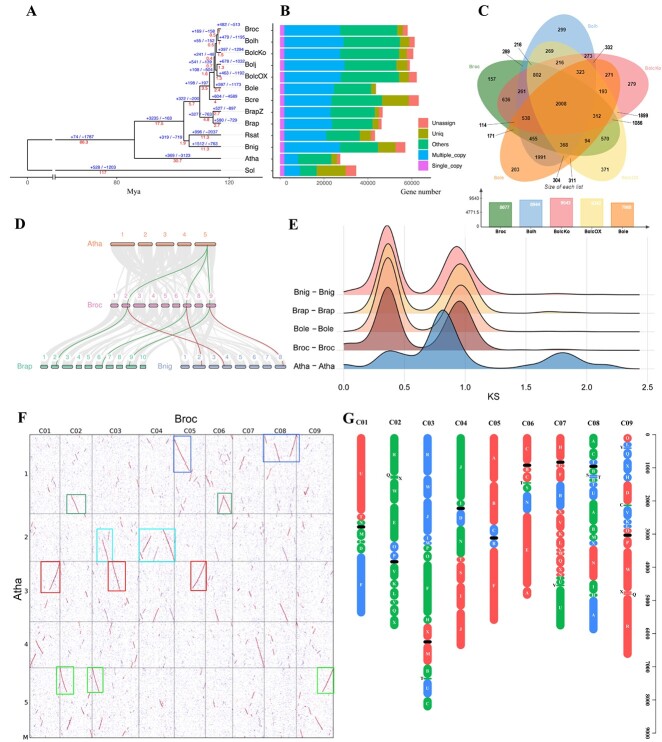
Comparative genomics analyses. **A** Phylogenetic tree of 13 plant species. The red numbers denote divergence time of each node (Mya, million years ago). The blue number on each branch of the tree denote the total number of expansion and contraction gene clusters. **B** Distribution of single-copy, multiple-copy, unique, and other orthologs in the 13 plant species. **C** Venn diagram representing the shared and unique gene cluster among five *Brassica* subspecies (Bop04-28-6, HDEM, Korso, TO1000, and OX-heart). Each number represents the number of gene clusters. **D** Synteny patterns between genomic regions from *A. thaliana*, *B. nigra*, *B. rapa*, and Bop04-28-6. This collinear relationship is highlighted by one syntenic set shown in green and red. **E** Distribution of synonymous substitution levels (*K*_s_) of syntenic paralogous genes (solid curves) after evolutionary rate correction. **F** Syntenic blocks between genomes. Dot plots of orthologs show a 1–3 chromosomal relationship between *A. thaliana* and Bop04-28-6. **G** Distribution of genomic blocks along the nine chromosomes of the Bop04-28-6 genome. Genome blocks were assigned to the subgenomes LF (red), MF1 (green), and MF2 (blue). Centromeres are shown as black ovals.

The high-quality reference genome for broccoli enables comparative genomics among Cruciferae species to investigate the genetic evolutionary basis of trait diversity. The *Brassica* species genomes for one *B. nigra*, two *B. rapa* (Chiifu and Z1) [12], and five *B. oleracea* (Korso, OX-heart, TO1000, HDEM, and JZS) and three Cruciferae species genomes of *A. thaliana* [39], *B. cretica* [40], and *R. sativus* [41], and one outgroup species genome were included for comparative genomic and phylogenomic analyses with the broccoli genome (Supplementary Data Table S3). OrthoVenn3 was used to identify putative orthologous gene clusters among annotated genes of the cruciferous species, based on pairwise sequence similarities. A total of 58 087 PCGs of the broccoli genome were grouped into 38 207 gene clusters. Compared with the other *Brassica* crops, 15 256 common gene clusters contained 248 618 genes, and 215 broccoli-specific clusters contained 1505 genes (Supplementary Data Table S4). Moreover, 2008 gene clusters contained 13 239 genes that were shared by all five *Brassica* species (Supplementary Data Table S4; Fig. 2A and B), and 289 gene clusters contained 997 genes clustered from two broccoli lines (BOP04-28-6 and HDEM).

**Table 1:** Assembly statistics of the broccoli genome.

Genome assembly	Number	Size
GC content		36.62%
Total contigs (bp)	552	613 792 861
Maximum contig length (bp)		49 006 907
Contig N50 (bp)	12	14 708 456
Contig N90 (bp)	50	2 802 620
Total scaffolds (bp)	478	613 800 261
Maximum scaffold length (bp)		79 934 553
Scaffold N50 (bp)	5	64 340 119
Scaffold N90 (bp)	9	52 555 196
Pseudochromosomes (bp)	9	576 183 561

Gene family contraction and expansion were detected in broccoli and the other seven species: BOP04-28-6, HDEM, Korso, TO1000, and OX-heart, Chiifu, *B. nigra*, and *A. thaliana* (Supplementary Data Fig. S3A). A total of 49 626 gene clusters were inferred from their most recent common ancestor using the Computational Analysis of gene Family Evolution (CAFE) tool, based on species divergence times and gene family clustering (Supplementary Data Table S4). CAFE analysis revealed 447 expansion gene families and 606 contraction gene families in the broccoli genome (Supplementary Data Fig. S3A). All of these families exhibited significant contractions and expansions (family-wide *P*-value ≤0.05) and were mainly assigned to macromolecule localization, peptide transport, and protein transport according to GO term enrichment analyses (Supplementary Data Fig. S3B; Supplementary Data Table S5).

Detailed investigation of the phylogenetic placement of broccoli based on 1923 single-copy gene families was conducted through phylogenetic analysis using the maximum likelihood method (Fig. 2A). The results showed that AA (*B. rapa* and *B. rapa* Z1), BB (*B. nigra*), and CC genomes (*B. oleracea* var. *botrytis*, Korso; *B. oleracea* var. *capitata,* OX-heart and JZS; *B. oleracea*, TO1000; *B. oleracea* var. *italica* HDEM and BOP04-28-6) were divided into different branches, and *B. cretica* was placed in the CC genome branch. The relationships between AA genome species and CC genome species, and between *B. nigra* and *R. sativus*, were relatively close (Fig. 2A). The divergence times were estimated using MCMCTree, with calibration. The estimated divergence time of the cruciferous species was 117 Mya, and *A. thaliana* and *Brassica* crops diverged at 30.7 Mya. Within the *B. oleracea* crops, the initially edible organs were loose leaves; the floret ball (Korso, HDEM, and BOP04-28-6) and leaf ball [Cap02-12 (V2) and OX-heart 923] diverged from TO1000 almost simultaneously, at 2 Mya (Fig. 2A). Such organ differentiation likely represented adaptations to the changeable climate and reproduction.

Based on the syntenic blocks detected between BOP04-28-6 and other plant genomes, the 4DTv and synonymous substitution rates of synonymous sites (*K*_s_) values of gene pairs were estimated to investigate evolutionary relationships and genome structural evolution, and two whole-genome duplication (WGD) events were detected (Fig. 2D–F; Supplementary Data Fig. S4E and F) [5, 42]. Segmental collinearity analysis showed that BOP04-28-6 had larger centromeres than HDEM (Fig. 2D; Supplementary Data Fig. S4G and H).

A direct comparison between the genomes identified some single-nucleotide polymorphisms (SNPs), insertions/deletions (InDels), and structural variants (SVs). SVs are more likely to cause phenotype changes than are SNPs. There are abundant structural variations between our genome and the other *B. oleracea* genomes, including insertions, deletions, duplications, and translocations (Supplementary Data Fig. S4H). In total, 1 941 862 SNPs, 683 392 InDels and 26 358 SVs were identified through direct genome comparison of BOP04-28-6 and HDEM using SVIM-ASM. Some SVs were located in the exon region, which affects 1795 PCGs (Supplementary Data Table S12). KEGG enrichment analysis was applied to these genes, in which the MAPK signaling pathway was found to be enriched (*P =* 0.0083; Supplementary Data Fig. S4I). GO term annotation indicated functional enrichment in diverse biological processes/systems including lipid biosynthesis, monosaccharide metabolic processes, the protein–DNA complex, ADP binding, and oxidoreductase activity (Supplementary Data Fig. S4J).

**Figure 3:**
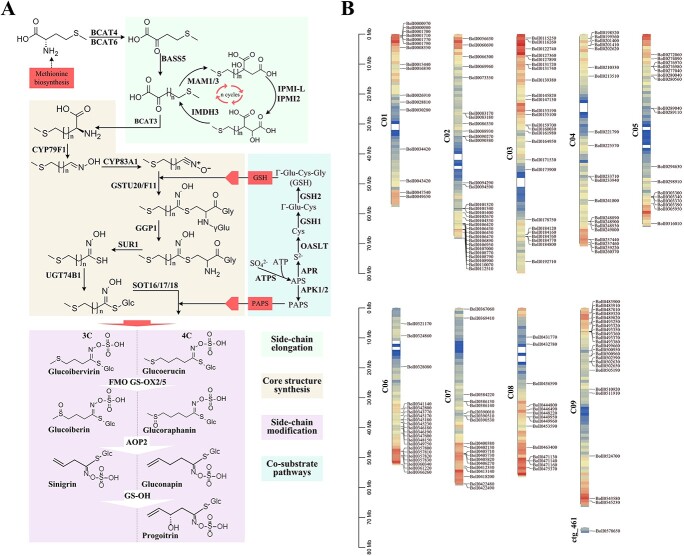
Aliphatic GSL biosynthesis pathway genes identification in broccoli. **A** Proposed biosynthetic pathway of aliphatic GSLs in broccoli. **B** Schematic representation of the broccoli chromosomes together with the positions of key genes involved in aliphatic GSL biosynthesis.

### Chromosome structure of the broccoli genome

A total of 58 087 genes were predicted from the BOP04-28-6 genome to examine the syntenic gene block arrangement in nine chromosomes. MCScanX was used to compare the gene block arrangement in three diploid species of U’s triangle [*B. rapa* (AA), *B. nigra* (BB), and *B. oleracea* (CC, 02-12)] with that of *A. thaliana* [13, 43, 44]. Relatively complete triplicated regions in broccoli were constructed and related to the 24 ancestral crucifer blocks (A–X) in *A. thaliana* [45]. Based on these syntenic relationships and their retained gene densities, the triplicated regions were divided into least fractionated (LF), medium fractionated (MF1), and most fractionated (MF2) subgenomes (Fig. 2G; Supplementary Data Table S6), and the same biased retention pattern of duplicated genes during diploidization was observed [43, 44]. In a gene-to-gene comparison, the LF subgenome comprised 24 769 genes, whereas MF1 and MF2 had 16 939 and 13 022 genes, respectively (Supplementary Data Table S6).

### Identification of glucosinolate biosynthetic pathway genes

GSL biosynthesis is divided into three independent stages: chain elongation, core structure formation, and secondary modifications [14, 23]. Based on the reported *A. thaliana* GSL biosynthesis genes [25, 26], the sequences were used as seeds to identify homologs in BOP04-28-6 and other *Brassica* varieties. We identified 121 GSL biosynthesis genes, 41 GSL degradation genes, 12 core transcription factors (TFs), and 8 GSL transporters. One or more gene copies and family members of these enzymes were identified among the 13 species (Supplementary Data Table S7). Almost all of the GSL biosynthetic pathway genes had multiple copies. The gene family consisting of *BoMAM*s, *BoCYP*s, *BoGGP1*, *BoSOT*s, *BoAPK*s, *BoGSH*s, *BoAOP*s, *BoGSL-OH*s, *BoESP*s, *BoNSP*s, *BoPCS*s, and *BoMY*s had two or more copies; the crucial TFs (BoMYB28, BoMYB29, BoMYB34, and BoMYB122) had two copies, and BoMYB51 had four copies. In our genome, the loci of *GS-ELONG*, *GS-AOP*, and *GS-OH* exhibited proximal and tandem duplication, which contributes to the diversity of GSLs. The *GS-ELONG* QTL is responsible for variation in GSL chain length [25, 46]; it contains *MAM* genes, with four *MAM* genes on chromosome 2 (BolI0088930, BolI0090270, BolI0108770, and BolI0108790) and two on chromosome 7 (BolI0405710 and BolI0405730). The *GS-AOP* QTL, which is responsible for secondary modification, contains *AOP* genes, including two on chromosome 2 (BolI0483900 and BolI0483910). Additionally, 13 genes were identified as *BoSOT18*: one on chromosome 1 (BolI0034420), seven on chromosome 6 (BolI0345170, BolI0345180, BolI0345230, BolI0346180, BolI0346190, BolI0357810, and BolI0357820), and five on chromosome 9 (BolI0500950, BolI0500960, BolI0502590, BolI0502630, and BolI0502650).

**Figure 4:**
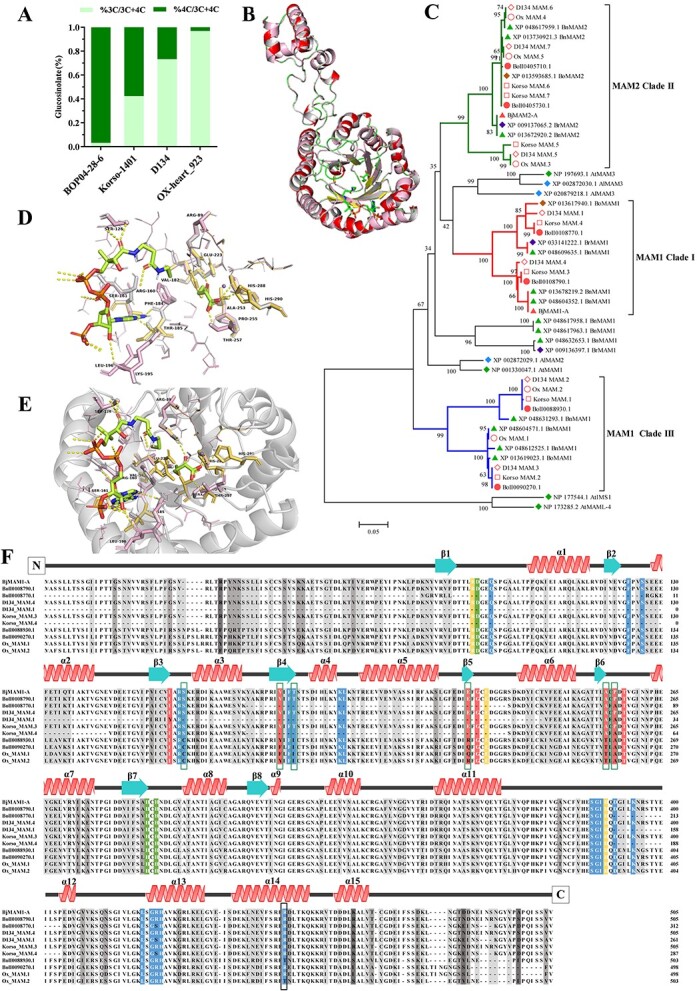
Function identification of *B. oleracea* MAMs. **A** Comparison of C3- and C4-glucosinolate content in four *Brassica* subspecies (BOP04-28-6, Korso-1401, D134, OX-heart_923). The C3-glucosinolate (C3-GSL; dark green) and C4-glucosinolate (C4-GSL; light green) profiles (shown as percentages) were determined in seedlings. **B** Comparison of 3D structures of BoMAM1, BjMAM1-A, and OX_MAM.1. The structures of BoMAM1 (α-helices and β-strands, gray), BjMAM1-A (α-helices and β-strands, rose), and OX_MAM.1 (α-helices, red and β-strands, yellow) monomers are superimposed. Conserved features, including the N-terminal α/β-barrel domain and the α-helical region that extends to form part of the CoA binding site, are indicated. **C** Phylogenetic analysis of MAMs from BOP04-28-6 (Bol), Korso-1401 (Korso), D134, OX-heart_923 (OX), *B. rapa* (field mustard; Br), *B. napus* (rape; Bn), *B. oleracea* (Bo), *A. thaliana* (thale cress; At), and *A. lyrata* subsp. *lyrata* (Al). Black numbers indicate the percentage of replicate trees in which the associated proteins clustered in the bootstrap test (1000 iterations). The evolutionary history was inferred using the neighbor-joining method. **D** Stereo view of the active site. Residues interacting with 4MTOB, CoA and Mn^2+^ (M) and surrounding the site are shown as stick models. **E** Stereo view of the substrate-binding pocket. Residues encompassing 4MTOB and CoA are shown as stick models with the β-strands forming the interior of the pocket shown as ribbons. **F** Multiple sequence alignment of BOP04-28-6, D134, OX-heart_923, and *B. juncea* MAMs. Secondary structure features corresponding to the structure of BoMAM1 (α-helices red, β-strands turquoise) are shown above the alignment. Residues in the metal-binding (green), catalytic (yellow), 2-oxo acid-binding (red), and CoA-binding (blue) sites are highlighted. Dark gray indicates regions of sequence difference and light gray indicates regions of sequence similarity.

**Figure 5:**
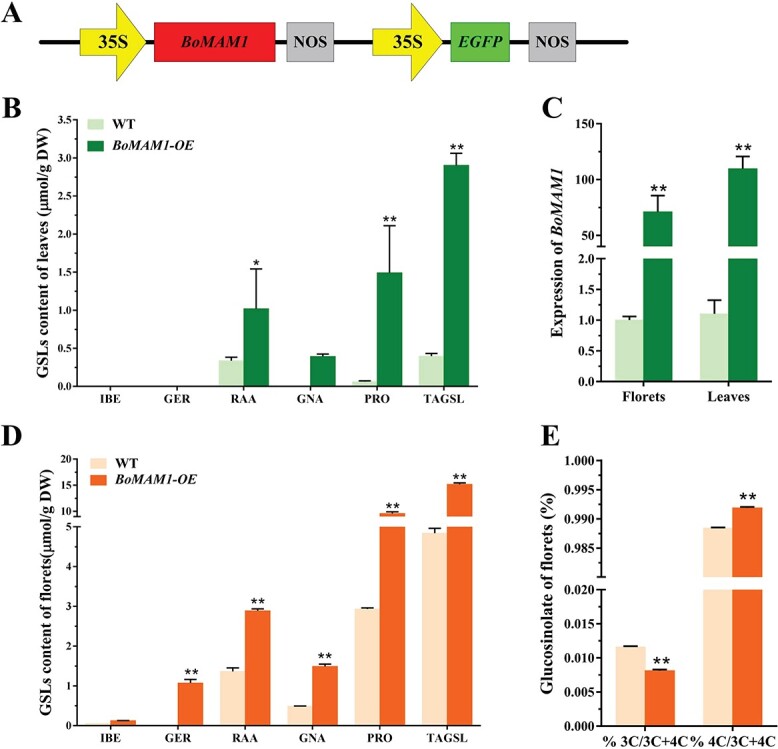
Overexpression of *BoMAM1* increased C4-GSL content and ratio. **A** Schematic diagram showing the constructs used for genetic transformation. **B**, **D** Aliphatic GSL content of *BoMAM1* overexpression lines and WT. **C** Relative expression of *BoMAM1* in overexpression lines and WT. **E** Comparison of C3- and C4-GSL content in *BoMAM1* overexpression lines and WT.

Amino acid sequence alignment and phylogenetic construction were conducted to demonstrate the functions of *BoMAM*s and *BoAOP*s (Fig. 4; Supplementary Data Fig. S5). The BoMAM enzymes belonged to the DRE-TIM metallolyase superfamily, according to gene sequence and structural comparisons; these enzymes catalyze carbon–carbon bond-forming reactions between acetyl-CoA and α-ketoacids. Phylogenetic analysis classified four *BoMAM*s as *BoMAM1* (BolI0088930, BolI0090270, BolI0108770, and BolI0108790) and two *BoMAM*s as *BoMAM2* (BolI0405710 and BolI0405730; Fig. 4C). In addition, BolI0108790 shares evolutionary ancestry and 97.23% sequence identity with BjMAM1_A (BjuA033169) [27], for which the protein structure was elucidated by a 2.1-Å-resolution X-ray crystal structure (Fig. 4B and F). According to the PROSITE database (http://prosite.expasy.org/), the conserved domain for carbon–carbon bond-forming reactions between acetyl-CoA and α-ketoacids has active-site residues including 3 metal-binding sites, 3 catalytic sites, 7 2-oxo acid binding sites, and 19 CoA binding sites (Fig. 4F) [36]. Amino acid sequence alignment revealed that the metal-binding and catalytic sites are better conserved than other sites. We hypothesized that BolI0108790 has the same catalytic function as BjMAM1_A. However, sequence alignment indicated the lack of a catalytic active site for BolI0108770, which is considered to be a pseudogene. Moreover, BolI0088930 and BolI0090270 have ~70% sequence identity with BjMAM1_A protein. Furthermore, variations in the acetyl-CoA and 2-oxo acid binding sites were observed, which indicates that these two genes have no catalytic function (Fig. 4F). Of the *BoAOP* genes, four were clustered as *AOP2* (BolI0090380, BolI0147130, BolI0483900, and BolI0483910). The 2-oxoglutarate/Fe(II)- dependent dioxygenase activity depends on two conserved domains, DIOX-N and 2OG-FeII_Oxy, at the N- and C-terminal regions (Supplementary Data Fig. S5B) [47]. In the conserved 2OG-FeII_Oxy domain of BoAOP2, four key active site residues were observed (His308, His356, Asp310, and Arg376). Sequence alignment suggested that only BolI0483910 has the same catalytic activity as BrAOPs (Supplementary Data Fig. S5) [47] .

### Characterization of glucosinolates and *MAM*s in *B. oleracea* varieties

Next, we investigated the underlying molecular mechanisms of GSL diversity in cultivated *B. oleracea* varieties. Four genomic sequences materials (broccoli, BOP04-28-6; cauliflower, Korso-1401; cabbage, D134; and OX-heart_923) were selected to contrast the profile ratio of GSLs (Supplementary Data Table S10). The results showed that BOP04-28-6 contained a higher C4-GSL content than C3-GSL; however, OX-heart_923 showed the inverse ratio, which indicated that the side chain elongation was the key factor for causing GSL diversity between BOP04-28-6 and OX-heart_923.

The evolutionary and biochemical foundation of the *BoMAM* gene family of *B. oleracea* crops plays a crucial role in GSL profile diversity. Among these four *Brassica* crop genomes, six (BOP04-28-6), seven (Korso-1401), seven (D134), and five (OX-heart_923) *MAM*s were identified. Phylogenetic tree analysis indicated that only single MAMs for D134 (D134_MAM.4) and Korso-1401 (Korso_MAM.3) were clustered with *BolI0108790_BoMAM1* and *BjMAM1-A*, which shared 97.23% homology with BjMAM1-A, with OX-heart missing the MAMs in clade I (Fig. 4C). Although the two OX-heart MAMs (*Ox MAM.1* and *Ox MAM.2*) were clustered with the clade III *BoMAM1* (BolI0088930.1 and BolI0090270.1) of BOP04-28-6, the mutation of active-site residues suggests that *Ox MAM.1* and *Ox MAM.2* cannot catalyze 4-methyl-thio-2-oxobutanoic acid (4MTOB).

To confirm this hypothesis, protein (BolI0108790_BoMAM1 and Ox_MAM.1) structure homology was examined using Swiss-Model (https://swissmodel.expasy.org/). Based on the MAM crystal structure from *B. juncea* (BjuMAM1-A), this protein in a homodimer contains two CoA, two 4-(methylsulfanyl)-2-oxobutanoic acid, two Mn^2+^ ligands, eight β-strands, and α-helices; the BolI0108790_BoMAM1 protein obtains only two Mn^2+^ ligands (Supplementary Data Fig. S6). To investigate the major residues for substrate binding, the 3D protein structure of BolI0108790_BoMAM1 was visualized using PyMOL [48, 49]. The active-site residues of BolI0108790_BoMAM1 were replaced to build the 3D protein model, and the protein surface of the substrate binding site was formed by a major portion of residues containing Val-182, Glu-223, Ala-253, and Pro-255 (Fig. 4D–F), which likely governed the substrate preference of MAMs [36]. The results indicated that the BolI0108790_BoMAM1 and its highly homologous MAMs have major catalysis ability for C4-GSLs, and the shortage of C4-GSLs in OX-heart_923 is due to the missing clade I MAM1 member and residue variation.

### Overexpression of *BoMAM1* induces C4-glucosinolate accumulation

To address the functional significance of *BoMAM1*, an *Agrobacterium*-mediated genetic transformation technique was used to construct a *BoMAM1* overexpression transgenic line, and BOP04-28-6 was transformed. Quantitative reverse-transcription polymerase chain reaction (qRT–PCR) was used to assess *BoMAM1* expression in the *BoMAM1-OE* line. A significantly high expression level was detected in florets and leaves (Fig. 5C). When the broccoli had grown to maturity, florets and leaves were sampled for GSL analysis. For C4-GSL, the gluconapin (GNA) and progoitrin (PRO) contents had the greatest improvement (2-fold) compared with WT florets. A 23-fold increase of PRO in *BoMAM1-OE* leaves was observed, as well as a 1-fold increase in florets and a 2-fold increase in leaf RAA concentrations in the *BoMAM1-OE* line (Fig. 5B and D). Glucoerucin (GER) accumulated in florets but was undetected in WTs; however, GNA was detected in *BoMAM1-OE* leaves (Fig. 5B and D). Notably, C4-GSL content showed a significant increase compared with the WT, and the ratio of C4-GSL was enhanced in florets and leaves of the *BoMAM1-OE* line (Fig. 5E).

### Integrated transcriptomic and metabolomic analyses for glucosinolate biosynthesis and transport

**Figure 6:**
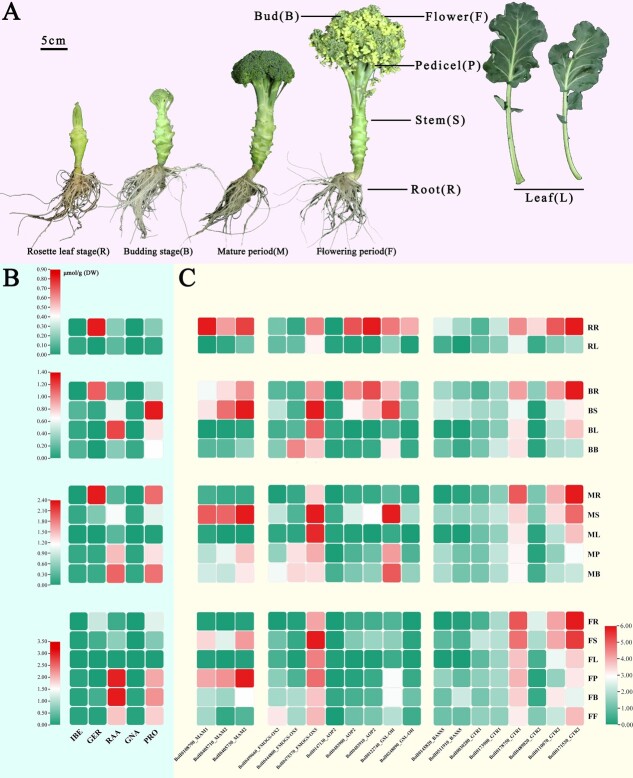
Aliphatic GSLs content and biosynthesis genes expression pattern. **A** Broccoli morphology in four growth periods. **B** Aliphatic GSL content of broccoli. **C** Gene expression profile of* MAMs*, *FMO GS-OXs*, *AOPs*, *GS-OHs*, *BASS5s*, and* GTRs* for aliphatic GSL biosynthesis and transport in different tissues in development phases in broccoli.

To obtain insights into the spatiotemporal distribution characteristics of GSLs in broccoli, we conducted high-performance liquid chromatography (HPLC) to detect GSLs, using sinigrin as the internal standard. The four growth stages (rosette leaf, budding, maturation, and flowering stages) were observed in different tissues (roots, stems, pedicels, leaves, buds, and flowers; Supplementary Data Table S8). Four aliphatic GSLs (IBE, GER, RAA, and PRO) were identified and quantified (Fig. 6B). Significant differences in GSL composition were observed among the different growing stages and tissues. The primary aliphatic short-chain GSL (C3–C4) for the C3-aliphatic GSL IBE was detected in all 17 samples; its synthesis was enhanced during the maturation and flowering stages, and it was enriched in the roots and three reproductive organs (pedicel, bud, and flower). For the C4-aliphatic GSLs GER and RAA, GER was not detected in leaves in any of the four growth stages, but accumulated massively in the roots. RAA, the metabolite of GER, was enriched in reproductive organs during the maturation and flowering stages and was detected in all 17 samples (Fig. 6B) [19, 23, 50]. Correlation analyses showed a significant negative correlation between GER and RAA contents, whereas IBE and RAA were strongly positively correlated (*P* < 0.01; Supplementary Data Fig. S7).

To investigate the molecular mechanism underlying GSL biosynthesis sites and transport properties, we performed transcriptome analysis of the broccoli developmental stages, based on the high-quality genome. Expression profiling analysis revealed that most genes involved in the GSL biosynthesis pathway exhibited tissue-specific expression (Supplementary Data Fig. S7 and Table S9). *BoMAM1* (BolI0108790), which is involved in side chain elongation, was highly expressed in the roots during the rosette leaf and budding stages. The core structure synthesis genes *BoCYP83A1* (BolI0233710), *BoGSTU20* (BolI0342800), *BoGGP1* (BolI0008350, BolI0418200), *BoUGT74B1* (BolI0280040), *BoSOT16* (BolI0357830), and *BoSOT18* (BolI0357820) also attained high expression levels in roots and other tissues, probably because this gene family performs more extensive functions in plants (Supplementary Data Fig. S7A). In contrast, three *BoFMO GS-OX* family genes showed the opposite expression pattern, with *BoFMO GS-OX2* (BolI0499660) and *BoFMO GS-OX5-1* (BolI0444800) highly expressed in reproductive tissues and *BoFMO GS-OX5-2* (BolI0475370) highly expressed in stems. Among these four multicopy *BoAOP2* genes, only one had catalytic activity that is highly expressed in the roots during the rosette leaf and budding stages (Fig. 6C). To investigate the biosynthesis and transport mechanisms, the expression profile of these six GSL transporter genes (*BoGTR*s) was analyzed. *BoGTR* genes were found to be highly expressed in roots and shoots at all stages (Fig. 6C).

### Identification of potential transcription factors involved in glucosinolate biosynthesis

**Figure 7:**
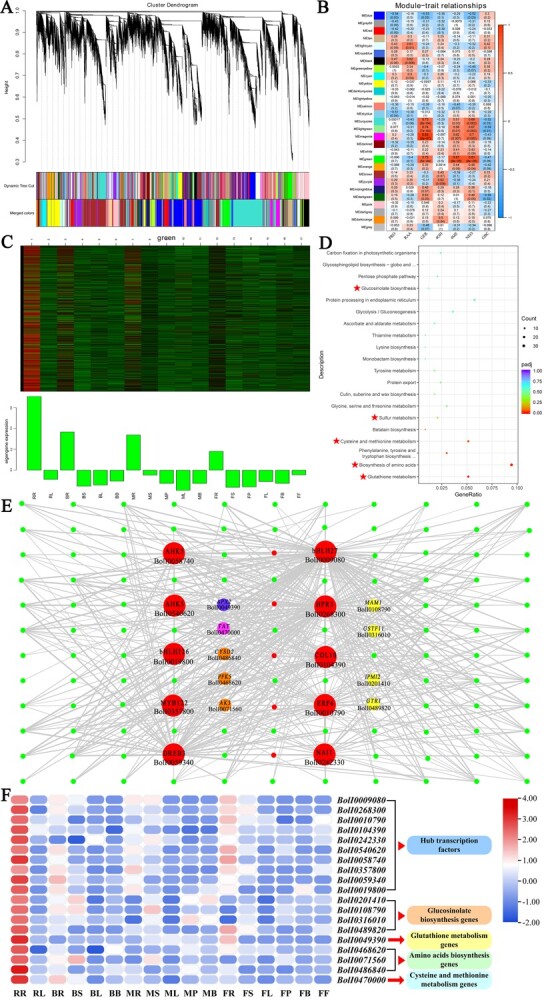
WGCNA of broccoli transcripts. **A** Dendrogram of genes based on co-expression network analysis. Each leaf in the tree corresponds to an individual gene. Twenty-nine modules, each associated with a different color, formed from the major tree branches. **B** Association between modules and GSL content. The color of each module is the same as that in **A**. The color scale indicates the correlation. Each cell contains the number of corresponding correlations and *P* values. **C** Heat maps and bar graphs of co-expressed genes in the MEgreen module. **D** KEGG pathway analysis of co-expressed genes in the MEred module. **E**, **F** Co-expression network and gene expression profile of the MEgreen module from WGCNA. Transcription factors with strong regulatory relationships (weight > 0.3) are indicated by red circles, the GLS biosynthesis genes, glutathione metabolism genes, amino acid biosynthesis genes and cysteine and methionine metabolism genes of the MEgreen module with strong regulatory relationships (weight > 0.3) to TFs are indicated by yellow, purple, orange, and peach circles.

Based on the gene expression profile, we conducted weighted gene co-expression network analysis (WGCNA) to examine the co-expression of GSL biosynthesis pathway genes. From the identified 61 440 genes across all samples, we extracted 28 040 variably expressed genes to build 29 co-expression modules (Fig. 7A and B). Among the 219 GSL biosynthesis-related genes, only 96 were clustered in the co-expression modules, and were scattered among 20 of the modules (Supplementary Data Table S11).

KEGG enrichment analysis was applied to the candidate modules (Supplementary Data Fig. S8). The pathways in which green module genes were significantly enriched contribute to GSL biosynthesis, such as the glutathione metabolism, amino acid biosynthesis, cysteine and methionine metabolism, sulfur metabolism, and glucosinolate biosynthesis pathways (Fig. 7D). Sixteen GSL biosynthesis-related genes were screened in the green module; the GSL side chain elongation gene *BoMAM1* (BolI0108790) was included. The rosette leaf stage root (RR) sample maintained a significantly positive correlation with the green module, and GER obtained the same correlation with the green module (Fig. 7C). In addition, modular genes were highly expressed in broccoli roots. These results further indicate the unusually high expression of *BoMAM1* in broccoli tissues.

Additionally, 112 TFs were screened from the green module, belonging to 31 TF families (Supplementary Data Table S11). Based on a weight/topological overlap matrix threshold of >0.3, we filtered out 15 hub TFs, four genes related to GSL biosynthesis, one gene related to glutathione metabolism, three genes related to amino acid biosynthesis, and one gene involved in cysteine and methionine metabolism. The co-expression network of the green module was constructed using Cytoscape (Fig. 7E). The 10 TFs (BobHLH27, BolI0009080; BoHPR3, BolI0268300; BoERF6, BolI0010790; BoCOL10, BolI0104390; BoNAI1, BolI0242330; BoAHK5, BolI0540620; BoAHK5, BolI0058740; BoMYB122, BolI0357800; BoDREB3, BolI0059340; and BobHLH126, BolI0019800) among the hub TFs were connected to key pathway genes (Fig. 7E; Supplementary Data Table S11). Notably, two hub TFs (BobHLH27 and BoHPR3) were connected to all four GSL biosynthesis genes (*BoMAM1*, *IPMI2,* BolI0201410; *GSTF11,* BolI0316010; and *GTR1*, BolI0489820). These two hub TFs not only connected to the GSL biosynthesis genes but also to one glutathione metabolism gene (*APX2*, BolI0049390), all three amino acid biosynthesis genes (*CYSD2*, BolI0486840; *PFK5*, BolI0468620; and *AK3*, BolI0071560), and one cysteine and methionine metabolism gene (*TAT*, BolI0470000).

## Discussion

Although many genomes of *B. oleracea* have been investigated, the taxonomic divergence of this family based on its diverse agronomic traits remains unclear. In addition, the profile specificity of GSLs in broccoli receives much attention, but requires clarification with regard to their hereditary basis. In this study, a chromosome-level high-quality diploid *B. oleracea* var. *italica* Plenck (broccoli, BOP04-28-6) genome was assembled, and the sequences anchored to nine pseudo-chromosomes, corresponding to the BB genome karyotype (2*n* = 2*x* =18). For the previous genome of this species, the assemblies of contig N50 and scaffold N50 and BUSCO values were lower than ours; specifically, the contig N50 size value of the broccoli assembly was 1.6-fold larger than that of HDEM (9.49 Mb; Table 1) [12]. The genome size showed slight differences among *B. oleracea* varieties, with the exception of TO1000, possibly due to the short reads used to estimate the genome size [1, 13].

Phylogenomic analysis of 1923 single-copy gene families was conducted using the maximum likelihood method. The AA, BB, and CC genomes were divided into different branches (Fig. 2A); the external physical characteristics were found to be strongly influenced by evolution. The commercial trait of the floret ball in *B. oleracea* was separated, and consistent species divergence status among taxa with AA, BB, and CC genomes was observed in some Brassicaceae family genome studies [4, 5, 8, 9]. WGDs are regarded as drivers of speciation, which can promote diversification [51]. After the split of the Brassiceae tribe from *Arabidopsis*, a recent and tribe Brassiceae-specific WGT occurred that greatly affected the genomic diversification of the extant *Brassica* species [1, 5, 13, 52]. In addition, the observation of 24 common syntenic genomic blocks and syntenic analyses of *Arabidopsis* and other Brassiceae taxa (Fig. 2G) [43], including phylogenetic trees, showed that the BB genomes are distantly related to the CC genomes and more closely related to the AA genomes [1, 44].

Genome mining is an effective means for discovering natural product biosynthetic pathways, facilitating their characterization. In *Brassica* and *Arabidopsis*, GSLs are the most prominent metabolite families, with highly relevant ecological and physiological functionality that provides their typical food flavors and protective effects against cancer [26, 53, 54]. Additionally, proximal duplication genes and tandem duplication genes were observed, which were due to the WGT event and gene block arrangement, indicating triplication or more compared with *Arabidopsis* genome genes. However, GSL biosynthetic pathway genes exhibit a scattered distribution, with small clusters (Fig. 3B). Gene duplications, particularly tandem genes and gene clusters, play important roles in the diversification of plant secondary metabolic pathways [55, 56]. This phenomenon suggests that these gene pairs participate in concerted functions or attain some form of complementary subfunctionalization among genes.

Based on side chain elongation and secondary modification of GSLs, >120 known GSL structures have been identified, and ~40 types of GSLs are present in *Arabidopsis*, primarily derived from Met and Trp. *Brassica rapa* was enriched in C4/C5-hydroxyalkyl-GSLs, which are catalyzed from sulfinyl-GSLs by AOPs [47, 57]. The high enrichment of C3-hydroxyalkyl-GSL is an important characteristic of the GSL profile in *B. juncea* [58]*.* However, in *B. oleracea* the GSL profile differs among subspecies, and higher RAA accumulation is observed in broccoli [17]. In addition, C3-hydroxyalkyl-GSL is enriched in cauliflower and cabbage [59, 60]. The individual genes involved in GSL side chain elongation and secondary modification contribute to this diversity. The molecular function of genes depends on multiple factors, including the plant species, accession, and allelic condition [50]. Four major loci have been found to control aliphatic GSL variation: *GS-ELONG*, *GS-OX*, *GS-AOP*, and *GS-OH* [19, 28, 61, 62]. In our genome, the *GS-ELONG* and *GS-AOP* loci showed proximal duplication and tandem duplication. The *GS-ELONG* QTL, which contains six *BoMAM* genes (BolI0088930, BolI0090270, BolI0108770, BolI0108790, BolI0405710, and BolI0405730), contributes to variable GSL chain length [25, 46]. Based on phylogenetic analysis sequence alignment, the profile and content of GSLs, as well as the 3D protein model of MAM1 amino acid sequences in different *Brassica* taxa, indicated that BolI0108790 is the key enzyme for C4-GSL biosynthesis (Fig. 4D–F) [27]. Similarly, four AOP2s (BolI0090380, BolI0147130, BolI0483900, and BolI0483910) were identified; only BolI0483910 holds a key active site with catalytic activity comparable to that of AOPs (Supplementary Data Fig. S5) [47]. These data provide valuable clues for understanding the mechanism of GSL diversity, given the high C4-GSL content. The identified genes are valuable targets for genetic improvement regarding the high nutritional value of the species. Thus, the diversification of the *B. oleracea* GSL profile is due to duplication and gene function conservation of the protein ligand site.

RNA-seq, GSL profiling, and content determination were performed for 17 samples among four growth stages (rosette leaf, budding, maturation, and flowering stages) for different tissues (roots, stems, pedicels, leaves, buds, and flowers) of BOP04-28-6 (Supplementary Data Table S8). The C3- and C4-GSLs showed an organizational difference in accumulation; C4-GSLs showed a negative correlation between GER and RAA. RAA is catalyzed from GER by BoFMO GS-OXs [19, 23, 50]. However, IBE and RAA were significantly positively correlated (*P* < 0.01), and IBE and RAA have the same amino acid side chain group, combined with the expression level of three *BoFMO GS-OXs* (Fig. 6C). Thus, the associated catalysis presents substrate specificity [63–66]. In addition, BoFMO GS-OX5 is more active during 3-(methylsulphinyl)propyl GSL catalysis. As another enzyme of amino acid side chain group diversity in *Brassica* [47], only one AOP2 (BolI0483910) exhibited catalytic activity that was highly expressed in roots during the rosette leaf and budding stages. Considering the tissue-specific expression of BoAOP2 (BolI0483910) and the tissue differential distribution of RAA and IBE, a shortage of substrates catalyzed by BoAOP2 may form hydroxyalkyl GSLs. As plant secondary metabolites, which have specific synthesis sites and are transported to other tissues, GSLs appear to be stored in edible parts or exhibit resistance to pests and diseases [67–70]. During the evolution of *B. oleracea*, conservation of the crucial catalytic active site of MAM1 ensured high RAA accumulation in broccoli. *MAM* family genes are involved in GSL biosynthesis at the first side chain elongation stage [34, 46], and gene expression at the beginning of GSL biosynthesis is susceptible to regulation [71]. In addition, *BoMAM1* (BolI0108790) was highly expressed in roots during the rosette leaf and budding stages, and GER accumulated in roots during the rosette leaf, budding, and maturation stages. The key enzyme BoMAM1 (BolI0108790) for C3- to C4-GSL synthesis is considered to be an indicator of the initial C4-GSL synthesis site. Furthermore, the negative correlation of enrichment between GER and RAA, as well as combined expression levels of FMO GS-OXs, and glucosinolate transporters (GTRs), indicates that GER was initially biosynthesized in roots, transported to reproductive tissues, and then catalyzed to RAA; this process resulted in the hydrolysis of products with anticancer activity, conferring health promotion benefits on broccoli.

Enzymatic reactions are crucial for GSL biosynthesis, and transcriptional regulation is the potential factor altering the expression of enzyme genes for GSL accumulation in different tissues [71]. In some biological processes, the mutual interaction and binding of TFs to gene promoters play key roles in integrating regulatory information. Many core TFs for GSL biosynthesis regulation were identified. Three MYBs (MYB28, MYB29, and MYB76) are central to aliphatic GSL synthesis and three (MYB34, MYB51, and MYB122) to indolic GSL synthesis [26, 72]. Among the bHLH subgroups, MYC2/bHLH06, MYC3/bHLH05, and MYC4/bHLH04, belonging to subgroup III, interact with MYBs and directly regulate GSL biosynthesis [73, 74]. WGCNA is an effective tool for investigating the key genes and TFs of target metabolites for large amounts of transcriptome data. Through WGCNA and module gene function enrichment analyses, the green module genes were found to be enriched during GSL biosynthesis and in the other pathways that contribute to GSL biosynthesis. The co-expression TFs of GSL biosynthesis genes were screened. BobHLH27 and BoHPR3 were found to be connected to all of the key pathway genes, indicating that these two TFs would play the important role in GSL biosynthesis. The bHLH TF belonging to BobHLH27, which was covered as an early network component with a role in pathogen and insect resistance, and its transcriptional activity are induced by methyl jasmonate [75]. Similarly, the key TFs (MYC2/MYC3/MYC4) of the jasmonic acid signaling pathway integrate environmental stresses and developmental signals to regulate plant growth and defense [76]. Moreover, BoHPR3 is the redundancy gene in the hydroxypyruvate-reducing system [77]; however, information on its transcriptional regulation function is lacking to date. BobHLH27 likely controls differential GSL accumulation in tissues to regulate expression of these four GSL biosynthesis gene, and it has a potential regulatory function in the other pathways that contribute to GSL biosynthesis. However, the enzymatic genes and co-expression TFs were highlighted using bioinformatics tools. Resolution of the gene function is necessary to verify the differential tissue accumulation and diversity of GSL profiles in tissues.

## Conclusions

A high-quality, chromosome-level genome assembly of broccoli was conducted using Illumina sequencing, PacBio SMRT, and Hi-C technology. Contig N50 reached 14.70 Mb, with an overall genome size of 613.79 Mb. Based on gene information for *B. oleracea* varieties and detailed transcriptome data, we revealed that BoMAM1 is the key enzyme contributing to diversification across *Brassica* species in terms of GSL structure and differential accumulation in broccoli tissues. A global view of the regulatory network of GSL biosynthesis in broccoli and many potential candidate genes was investigated to determine whether GSL biosynthesis is regulated by multiple TFs in broccoli. This study provides valuable resources for exploring the evolution of *Brassica* crops, which is valuable for future genetic studies and nutritive component applications. The presented high-quality broccoli genome is also an important resource for molecular breeding.

## Materials and methods

### Plant materials and DNA and RNA isolation

An advanced-generation inbred line of *B. oleracea* L var. *italica* (BOP04-28-6, broccoli) was selected for whole-genome sequencing. This line has excellent agronomic traits and strong RAA biosynthesis ability, and the hydrolysis product of RAA confers high anti-cancer activity. The seedlings were cultivated in a 50-well nursery site and then planted in the greenhouse (Yunyuan base of Hunan Agriculture University) when they had grown to four true leaves (on 28 September 2020). The young leaves were collected for genomic DNA extraction and genome determination at the rosette leaf stage. DNA was extracted by the phenol–chloroform extraction protocol. The roots, stems, pedicels, leaves, buds, and flowers at rosette leaf stage, budding stage, mature period and flowering period were sampled for RNA-seq and GSL content measurement. Following the manufacturer’s instructions for TRIzol reagent (Invitrogen, USA), total RNA was extracted and pooled for sequencing. Seven-day-old seedlings of BOP04-28-6, Korso-1401, D134, and OX-heart_923 were cultivated in a phytotron (22°C, 60% humidity, 12 h light/12 h dark, Hunan Agricultural University) and collected for GSL content measurement.

### Estimation of genome size

Based on *k*-mer distribution, a genomic survey was conducted to estimate the genome size, GC content, homozygosity status, and duplication content. A library construction kit (Illumina) was used to construct a short insert size (350 bp) library, and the Illumina HiSeq 6000 platform was used for sequencing. Jellyfish [78] was used to determine the distribution of *k*-mer values based on the generated ~50× high-quality reads, and GenomeScope [79] was used to estimate genome size.

### Genome sequencing, assembly, and quality evaluation

Using AMPure PB Magnetic Beads (Pacific Biosciences), DNA concentration, damage repair, end repair, ligation of hairpin adapters, and template purification were conducted to prepare the SMRTbell template. Then, the constructed DNA libraries was carried out on the PacBio Sequel platform. CCS (https://github.com/PacificBiosciences/ccs) was used to filter the high-precision HiFi reads, based on subreads. *De novo* assembly of the PacBio HiFi reads was performed using Hifiasm to obtain contig sequences.

Genome integrity was evaluated using BUSCO (Benchmarking Universal Single-Copy Orthologs: http://busco.ezlab.org/) and CEGMA (Core Eukaryotic Genes Mapping Approach: http://korflab.ucdavis.edu/datasets/cegma/). The integrity of the assembled genome was evaluated by BUSCO and CEGMA. BWA software (http://bio-bwa.sourceforge.net/) was used to analyze the alignment rate, extent and depth distribution of reads covering the genome, to evaluate the accuracy of assembly.

### Hi-C sequencing and assistant assembly

Hi-C library is prepared by the protocol, crosslink DNA, cut with restriction enzyme, fill ends and mark with biotin, ligate, purify and shear DNA, pull down biotin, and sequencing using paired-ends on Illumina HiSeq-2500 platform (PE 125bp). Hi-C data was assembled by ALLHiC, and through 5 steps (pruning, partition, rescue, optimization, building) to own chromosomal level assembly. ALLHiC could handle both the assembly of simple genomes and the assembly of complex genomes (high heterozygosity, polyploidy).

### RNA sequencing and analysis

Total RNA was used as input material. Briefly, mRNA was purified using poly-T oligo-attached magnetic beads. Stranded RNA libraries were constructed and sequenced by the IlluminaNovaseq platform. The clean data was filter out and then mapped onto this broccoli genome. The mapped reads were assembled by StringTie (vl.3.3b). We used FeatureCounts vl.5.0-p3 to count the read numbers mapped to each gene. The lengths of the genes and read counts were used to calculate the FPKM of each gene. The DESeq2 R package (1.20.0) was used to analyze the differential expression of two groups. DEGs were assigned using an adjusted *P*-value of <0.05. We used the clusterProfiler R package to analyze GO enrichment; significantly enriched GO terms were identified using a corrected *P*-value <0.05. We used the clusterProfiler R package to test the statistical enrichment of KEGG pathways.

To analyze genes involved in GSL biosynthesis of different broccoli tissues for four developmental stages, WGCNA was performed using the R package. To construct an adjacency matrix we used 7 as the soft thresholding power, and all DEGs were hierarchically clustered by TOM similarity. The first principal component was used to convert the genes in different colored modules to module eigengenes. The GSL contents of different broccoli tissues for four developmental stages were correlated with the eigengenes of each module to find the key module associated with GSL biosynthesis. Hub genes were identified based on the threshold of weight/topological overlap matrix (TOM) > 0.3.

### Genome annotation

Homologous protein sequences were downloaded from Ensembl and TAIR (Swiss-Prot and TAIR10). Diamond was used for alignment to the genome and protein sequences. To predict the gene structure of the protein region, the matching proteins were aligned to the homologous genome sequences*.* Augustus (v3.2.3), GeneMark-ES, GlimmerHMM (v3.04), and SNAP (2013-11-29) were used for automated gene prediction. The *ab initio* gene prediction models were trained using the high-quality gene models from the PASA assembly using RNA-seq data. Tandem repeats were extracted using Tandem Repeats Finder (http://tandem.bu.edu/trf/trf.html). And ab initio prediction built *de novo* repetitive elements database by LTR_FINDER, RepeatScout (http://www.repeatmasker.org/), RepeatModeler (http://www.repeatmasker.org/RepeatModeler.html) with default parameters. Gene functions were assigned according to the best match by aligning the protein sequences to Swiss-Prot using BLASTP (with a threshold of E-value ≤1e^−5^) and EggNOG (http://eggnog-mapper.embl.de/). We searched ProDom, PRINTS, Pfam, SMRT, PANTHER, and PROSITE to annotate the motifs and domains by InterProScan70 (v5.31). The protein functions predicted by database searches, including KEGG, GO, Swiss-Prot, and National Center for Biotechnology Information non-redundant. tRNAscan-SE (http://lowelab.ucsc.edu/tRNAscan-SE/) was used to predict tRNA. For rRNAs are highly conserved, we choose relative species’ rRNA sequences as references. BLAST was used to predict rRNA sequences. Other ncRNAs were identified by searching against the Rfam database with default parameters using the infernal software (http://infernal.janelia.org/).

### Comparative genomic and evolutionary analyses

Orthologous relationships between genes of *A. thaliana*, *B. nigra*, *B. oleracea* var. *botrytis* (Korso), *B. oleracea* var. *capitata* (OX-heart), *B. oleracea* var. *italia* (HDEM), and *B. rapa* (Z1) were inferred using all-against-all protein sequence similarity searches with OrthoFinder. For each gene family, Muscle was used for alignment, Gblocks was used to ambiguously align positions, and IQ-TREE 1.6.12 was used to construct the tree. MCMCTree (http://abacus.gene.ucl.ac.uk/software/paml.html) was used to calculate the divergence times between species and was implemented in PAML. The likelihood model originally implemented in the software package CAFE (http://sourceforge.net/projects/cafehahnlab/) was used to identify the gene families expansion or contraction.

### Evolution and expression analysis of key candidate genes

Based on the described gene function and its genome information, the *MAM*s and *AOP*s of broccoli were chosen for further analysis. BLASTP and HMMER were used to identify the homologous genes in other species. The phylogenetic trees and sequence alignment were constructed using MEGA11.

### Glucosinolate analysis

According to a previously described method [80], GSLs were extracted and quantified using the following steps. Briefly, the freeze-dried sample powder was soaked in 70% methanol with 100 μl of sinigrin (5 mmol/l, internal standard) for 20 min in a 75°C water bath, the cooling mixture added with barium acetate and then for centrifugation. We collected the supernatant and re-extracted the residues. GSLs were catalyzed by sulfatase to convert them to desulfated GSLs in a DEAE–Sephadex A-25 column, and the aqueous solution of desulfated GSLs was analyzed by HPLC (Agilent, CA, USA). The following linear gradient program at 30°C was performed: 0–20% methanol, 20 min; 20–30%, 5 min; 10 min and 3 min for isocratic elution, 40% and 90%; individually, with a 1 ml/min flow rate. Desulfated GSLs were detected with a diode-array detector (229 nm). According to the fingerprint of desulfated GSL retention time by HPLC–MS, GSLs were characterized as desulfated GSLs and the content was measured (expressed in micromoles per gram).

### Broccoli *BoMAM1* genetic transformation

Broccoli seedlings were transformed with *Agrobacterium tumefaciens* strain LBA4404. The coding sequence of *BoMAM1* was cloned. *BoMAM1* was inserted into pCAMBIA1301 by homologous recombination and expression was driven by the CaMV 35S promoter. The recombinant plasmid pCAMBIA1301-*BoMAM1* was used for genetic transformation, and hygromycin was used to select stable transformants.

### Quantitative real-time RT–PCR analysis

Total RNA was reverse-transcribed into cDNA by HiScript II Q RT SuperMix for qPCR (+gDNA wiper; Vazyme, China). The cDNA samples were used to prepare the qPCR reaction solution by using an AceQ qPCR SYBR Green Master Mix Kit (Vazyme, China). On the basis of the base sequence of genes, specific primers were designed at the ends of the open reading frame: *BoMAM1*-F: CCCTACCACCAGTTCCAACA; *BoMAM1*-R: TCTTGTCGGGGAGCTTGTTC; Actin-F: ATGGCTGAGGCTGATGACATTC; Actin-R: AAGGTCGAGACGGAGGATGG.

## Acknowledgements

This work was supported by the National Key Research and Development Program of China (2022YFF1003000), the National Natural Science Foundation of China (32372682, 32272747, 32072585, 32072568), the International Cooperation Projects of National Key R&D Program of China (2022YFE0108300), the Graduate Research Innovation Project of Hunan (2023XC103), and the innovation and entrepreneurship training program for college students (S202310537006X). We thank Qian Liu (1345595692@qq.com) for the methodology and validation of the comparative genomic analysis.

## Author contributions

Q.Y.W. and S.X.M. contributed equally to the work. S.X.M., Q.Y.W., and J.W.W. wrote the original draft and conceptualization; S.X.M., Y.S.W., H.P.H., J.L., X.C., L.H.H., and Y.X.T. performed data curation and investigation; Y.S.W. and J.H.Z. provided methodology and validation; Q.Y.W. and K.H. reviewed and edited the writing and acquired funding.

## Data availability

All raw sequencing data generated in this study have been deposited in figshare (https://figshare.com/) with the DOI number 10.6084/m9.figshare.24935037. Sequences of other species involved in this study were downloaded from the NCBI database (https://www.ncbi.nlm.nih.gov/), Brassicaceae Database (BRAD, http://brassicadb.cn), and the related references.

## Conflict of interest statement

No conflict of interest is declared.

## Supplementary data

Supplementary data are available at *Horticulture Research* online.

## Supplementary Material

Web_Material_uhae063

## References

[1] Isobel AP , Haibao T, Stephen JR. et al. Transcriptome and methylome profiling reveals relics of genome dominance in the mesopolyploid *Brassica oleracea*. Genome Biol. 2014;15:R77 24916971 10.1186/gb-2014-15-6-r77 PMC4097860

[2] NagaharoU . Genome analysis in *Brassica carinata* with special reference to the experimental formation of *Brassica napus* and peculiar mode of fertilization. Jpn J Bot. 1935;7:389–452

[3] Paritosh K , Yadava SK, Singh P. et al. A chromosome-scale assembly of allotetraploid *Brassica juncea* (AABB) elucidates comparative architecture of the A and B genomes. Plant Biotechnol J. 2021;19:602–14 33073461 10.1111/pbi.13492 PMC7955877

[4] Song X , Wei Y, Xiao D. et al. *Brassica carinata* genome characterization clarifies U's triangle model of evolution and polyploidy in *Brassica*. Plant Physiol. 2021;186:388–406 33599732 10.1093/plphys/kiab048 PMC8154070

[5] Waminal NE , Perumal S, Lee J. et al. Repeat evolution in *Brassica rapa* (AA), *B. oleracea* (CC), and *B. napus* (AACC) genomes. Plant Breed Biotechnol. 2016;4:107–22

[6] He Z , Ji R, Havlickova L. et al. Genome structural evolution in *Brassica* crops. Nat Plants. 2021;7:757–65 34045706 10.1038/s41477-021-00928-8

[7] Mabry ME , Turner SD, Gallagher EY. et al. The evolutionary history of wild, domesticated, and feral *Brassica oleracea* (Brassicaceae). Mol Biol Evol. 2021;38:4419–34 34157722 10.1093/molbev/msab183 PMC8476135

[8] Guo N , Wang S, Gao L. et al. Genome sequencing sheds light on the contribution of structural variants to *Brassica oleracea* diversification. BMC Biol. 2021;19:93 33952264 10.1186/s12915-021-01031-2 PMC8097969

[9] Lv H , Wang Y, Han F. et al. A high-quality reference genome for cabbage obtained with SMRT reveals novel genomic features and evolutionary characteristics. Sci Rep. 2020;10:12394 32709963 10.1038/s41598-020-69389-x PMC7381634

[10] Cai X , Wu J, Liang J. et al. Improved *Brassica oleracea* JZS assembly reveals significant changing of LTR-RT dynamics in different morphotypes. Theor Appl Genet. 2020;133:3187–99 32772134 10.1007/s00122-020-03664-3

[11] Sun D , Wang C, Zhang X. et al. Draft genome sequence of cauliflower (*Brassica oleracea* L. var. *botrytis*) provides new insights into the C genome in *Brassica* species. Hortic Res. 2019;6:82 31645943 10.1038/s41438-019-0164-0 PMC6804732

[12] Belser C , Istace B, Denis E. et al. Chromosome-scale assemblies of plant genomes using nanopore long reads and optical maps. Nat Plants. 2018;4:879–87 30390080 10.1038/s41477-018-0289-4

[13] Liu S , Liu Y, Yang X. et al. The *Brassica oleracea* genome reveals the asymmetrical evolution of polyploid genomes. Nat Commun. 2014;5:3930 24852848 10.1038/ncomms4930 PMC4279128

[14] Mao S , Wang J, Wu Q. et al. Effect of selenium-sulfur interaction on the anabolism of sulforaphane in broccoli. Phytochemistry. 2020;179:112499 32980712 10.1016/j.phytochem.2020.112499

[15] Wang J , Mao S, Wu Q. et al. Effects of LED illumination spectra on glucosinolate and sulforaphane accumulation in broccoli seedlings. Food Chem. 2021;356:129550 33819785 10.1016/j.foodchem.2021.129550

[16] Peñas E , Zielińska D, Gulewicz P. et al. Vitamin C, phenolic compounds and antioxidant capacity of broccoli florets grown under different nitrogen treatments combined with selenium. *Polish J Food Nutr Sci*. 2018;68:179–86

[17] Li Z , Zheng S, Liu Y. et al. Characterization of glucosinolates in 80 broccoli genotypes and different organs using UHPLC-triple-TOF-MS method. Food Chem. 2021;334:127519 32721832 10.1016/j.foodchem.2020.127519

[18] Peter R , Kathy F, Gary W. et al. 7-Methylsulfinylheptyl and 8-methylsulfinyloctyl isothiocyanates from watercress are potent inducers of phase II enzymes. Carcinogenesis. 2000;21:1983–8 11062158 10.1093/carcin/21.11.1983

[19] Li J , Hansen BG, Ober JA. et al. Subclade of flavin-monooxygenases involved in aliphatic glucosinolate biosynthesis. Plant Physiol. 2008;148:1721–33 18799661 10.1104/pp.108.125757 PMC2577257

[20] Nakagawa K , Umeda T, Higuchi O. et al. Evaporative light-scattering analysis of sulforaphane in broccoli samples: quality of broccoli products regarding sulforaphane contents. J Agric Food Chem. 2006;54:2479–83 16569031 10.1021/jf051823g

[21] Cartea ME , Velasco P. Glucosinolates in *Brassica* foods: bioavailability in food and significance for human health. Phytochem Rev. 2008;7:213–29

[22] Alumkal JJ , Slottke R, Schwartzman J. et al. A phase II study of sulforaphane-rich broccoli sprout extracts in men with recurrent prostate cancer. Investig New Drugs. 2015;33:480–9 25431127 10.1007/s10637-014-0189-z PMC4390425

[23] Sonderby IE , Geu-Flores F, Halkier BA. Biosynthesis of glucosinolates—gene discovery and beyond. Trends Plant Sci. 2010;15:283–90 20303821 10.1016/j.tplants.2010.02.005

[24] Brown AF , Yousef GG, Reid RW. et al. Genetic analysis of glucosinolate variability in broccoli florets using genome-anchored single nucleotide polymorphisms. Theor Appl Genet. 2015;128:1431–47 25930056 10.1007/s00122-015-2517-x

[25] Harun S , Abdullah-Zawawi MR, Goh HH. et al. A comprehensive gene inventory for glucosinolate biosynthetic pathway in *Arabidopsis thaliana*. J Agric Food Chem. 2020;68:7281–97 32551569 10.1021/acs.jafc.0c01916

[26] Simon M , Tamara G. Regulation of glucosinolate biosynthesis. J Exp Bot. 2020;72:70–91 10.1093/jxb/eraa479 33313802

[27] Kumar R , Lee SG, Augustine R. et al. Molecular basis of the evolution of methylthioalkylmalate synthase and the diversity of methionine-derived glucosinolates. Plant Cell. 2019;31:1633–47 31023839 10.1105/tpc.19.00046 PMC6635866

[28] Kroymann J , Textor S, Tokuhisa JG. et al. A gene controlling variation in *Arabidopsis* glucosinolate composition is part of the methionine chain elongation pathway. Plant Physiol. 2001;127:1077–88 11706188 PMC129277

[29] Kroymann J , Donnerhacke S, Schnabelrauch D. et al. Evolutionary dynamics of an *Arabidopsis* insect resistance quantitative trait locus. Proc Natl Acad Sci USA. 2003;100:14587–92 14506289 10.1073/pnas.1734046100 PMC304123

[30] Kitainda V , Jez JM. Structural studies of aliphatic glucosinolate chain-elongation enzymes. Antioxidants. 2021;10:1500 34573132 10.3390/antiox10091500 PMC8468904

[31] Liu Z , Hammerlindl J, Keller W. et al. *MAM* gene silencing leads to the induction of C3 and reduction of C4 and C5 side-chain aliphatic glucosinolates in *Brassica napus*. Mol Breeding. 2011;27:467–78

[32] Li Z , Zhang C, Cai Q. et al. Identification of *MAM1s* in regulation of 3C glucosinolates accumulation in allopolyploid *Brassica juncea*. Hortic Plant J. 2020;6:409–18

[33] Li Y , Tang X, Hirai MY. et al. Response of aliphatic glucosinolate biosynthesis to signaling molecules in MAM gene knockout mutants of *Arabidopsis*. Plant Biotechnol. 2013;30:403–6

[34] Zhang J , Wang H, Liu Z. et al. A naturally occurring variation in the *BrMAM-3* gene is associated with aliphatic glucosinolate accumulation in *Brassica rapa* leaves. Hortic Res. 2018;5:69 30534387 10.1038/s41438-018-0074-6 PMC6269504

[35] de Kraker J-W , Gershenzon J. From amino acid to glucosinolate biosynthesis: protein sequence changes in the evolution of methylthioalkylmalate synthase in *Arabidopsis*. Plant Cell. 2011;23:38–53 21205930 10.1105/tpc.110.079269 PMC3051243

[36] Kumar G , Johnson JL, Frantom PA. Improving functional annotation in the DRE-TIM metallolyase superfamily through identification of active site fingerprints. Biochemistry. 2016;55:1863–72 26935545 10.1021/acs.biochem.5b01193

[37] Zhang L , Cai X, Wu J. et al. Improved *Brassica rapa* reference genome by single-molecule sequencing and chromosome conformation capture technologies. Hortic Res. 2019;6:124 31728199 10.1038/s41438-019-0210-y PMC6851084

[38] Sampath P , Koh CS, Jin L. et al. A high-contiguity *Brassica nigra* genome localizes active centromeres and defines the ancestral *Brassica* genome. Nat Plants. 2020;6:929–41 32782408 10.1038/s41477-020-0735-y PMC7419231

[39] David S , Christopher W, Philippe L. et al. The *Arabidopsis* information resource (TAIR): gene structure and function annotation. Nucleic Acids Res. 2007;36:D1009–14 17986450 10.1093/nar/gkm965 PMC2238962

[40] Kioukis A , Michalopoulou VA, Briers L. et al. Intraspecific diversification of the crop wild relative *Brassica cretica* Lam. Using demographic model selection. BMC Genomics. 2020;21:48 31937246 10.1186/s12864-019-6439-x PMC6961386

[41] Hee-Ju Y , Seunghoon B, Young-Joon L. et al. The radish genome database (RadishGD): an integrated information resource for radish genomics. Database (Oxford). 2019;2019:baz009 30722041 10.1093/database/baz009 PMC6361821

[42] Franzke A , Lysak MA, Al-Shehbaz IA. et al. Cabbage family affairs: the evolutionary history of Brassicaceae. Trends Plant Sci. 2011;16:108–16 21177137 10.1016/j.tplants.2010.11.005

[43] Feng C , Mandáková T, Jian W. et al. Deciphering the diploid ancestral genome of the mesohexaploid *Brassica rapa*. Plant Cell. 2013;25:1541–54 23653472 10.1105/tpc.113.110486 PMC3694691

[44] Paritosh K , Pradhan AK, Pental D. A highly contiguous genome assembly of *Brassica nigra* (BB) and revised nomenclature for the pseudochromosomes. BMC Genomics. 2020;21:887 33308149 10.1186/s12864-020-07271-w PMC7731534

[45] Schranz ME , Lysak MA, Mitchell-Olds T. The ABC's of comparative genomics in the Brassicaceae: building blocks of crucifer genomes. Trends Plant Sci. 2006;11:535–42 17029932 10.1016/j.tplants.2006.09.002

[46] Das B . Glucosinolate biosynthesis: role of MAM synthase and its perspectives. Biosci Rep. 2021;41:10 10.1042/BSR20211634 PMC8490860 34545928

[47] Zhang J , Liu Z, Liang J. et al. Three genes encoding AOP2, a protein involved in aliphatic glucosinolate biosynthesis, are differentially expressed in *Brassica rapa*. J Exp Bot. 2015;66:6205–18 26188204 10.1093/jxb/erv331 PMC4588880

[48] Delano WL . The PyMol molecular graphics system. Proteins. 2002;30:442–54

[49] Rigsby REP , Alison B. Using the PyMOL application to reinforce visual understanding of protein structure. Biochem Mol Biol Educ. 2016;44:433–7 27241834 10.1002/bmb.20966

[50] Augustine R , Bisht NC. Regulation of glucosinolate metabolism. From model plant *Arabidopsis thaliana* to *Brassica* crops. In: Mérillon JM, Ramawat K, eds. Glucosinolates. Springer: Cham, 2017,163–99

[51] Tank DC , Eastman JM, Pennell MW. et al. Nested radiations and the pulse of angiosperm diversification: increased diversification rates often follow whole genome duplications. New Phytol. 2015;207:454–67 26053261 10.1111/nph.13491

[52] Lysak MA , Koch MA, Pecinka A. et al. Chromosome triplication found across the tribe Brassiceae. Genome Res. 2005;15:516–25 15781573 10.1101/gr.3531105 PMC1074366

[53] Katz E , Nisani S, Chamovitz DA. Indole-3-carbinol: a plant hormone combatting cancer. F1000 Res. 2018;7:689 10.12688/f1000research.14127.1 PMC5989150 29904587

[54] Ishida M , Hara M, Fukino N. et al. Glucosinolate metabolism, functionality and breeding for the improvement of Brassicaceae vegetables. Breed Sci. 2014;64:48–59 24987290 10.1270/jsbbs.64.48 PMC4031110

[55] Xu Z , Li Z, Ren F. et al. The genome of *Corydalis* reveals the evolution of benzylisoquinoline alkaloid biosynthesis in Ranunculales. Plant J. 2022;111:217–30 35476217 10.1111/tpj.15788 PMC7614287

[56] Xu Y , Zhang H, Zhong Y. et al. Comparative genomics analysis of bHLH genes in cucurbits identifies a novel gene regulating cucurbitacin biosynthesis. Hortic Res. 2022;9:uhac038 35184192 10.1093/hr/uhac038 PMC9071377

[57] Francisco M , Velasco P, Moreno DA. et al. Cooking methods of *Brassica rapa* affect the preservation of glucosinolates, phenolics and vitamin C. Food Res Int. 2010;43:1455–63

[58] Kim HW , Ko HC, Baek HJ. et al. Identification and quantification of glucosinolates in Korean leaf mustard germplasm (*Brassica juncea* var. *integrifolia*) by liquid chromatography–electrospray ionization/tandem mass spectrometry. Eur Food Res Technol. 2016;242:1479–84

[59] Cabello-Hurtado F , Gicquel M, Esnault M-A. Evaluation of the antioxidant potential of cauliflower (*Brassica olera*cea) from a glucosinolate content perspective. Food Chem. 2012;132:1003–9

[60] Park S , Valan Arasu M, Lee MK. et al. Quantification of glucosinolates, anthocyanins, free amino acids, and vitamin C in inbred lines of cabbage (*Brassica oleracea* L.). Food Chem. 2014;145:77–85 24128451 10.1016/j.foodchem.2013.08.010

[61] Jensen LM , Kliebenstein DJ, Meike B. Investigation of the multifunctional gene AOP3 expands the regulatory network fine-tuning glucosinolate production in *Arabidopsis*. Front Plant Sci. 2015;6:762 26442075 10.3389/fpls.2015.00762 PMC4585220

[62] Mithen R , Clarke J, Lister C. et al. Genetics of aliphatic glucosinolates. III. Side chain structure of aliphatic glucosinolates in *Arabidopsis thaliana*. Heredity. 1995;74:210–5

[63] Agerbirk N , Olsen CE. Glucosinolate structures in evolution. Phytochemistry. 2012;77:16–45 22405332 10.1016/j.phytochem.2012.02.005

[64] Jing L , Kristiansen KA, Hansen BG. et al. Cellular and subcellular localization of flavin-monooxygenases involved in glucosinolate biosynthesis. J Exp Bot. 2011;62:1337–46 21078824 10.1093/jxb/erq369

[65] Giamoustaris A , Mithen R, Genetics of aliphatic glucosinolates. IV. Side-chain modification in *Brassica oleracea*. Theor Appl Genet. 1996;93:1006–10 24162437 10.1007/BF00224105

[66] Kliebenstein DJ , Kroymann J, Brown P. et al. Genetic control of natural variation in *Arabidopsis* glucosinolate accumulation. Plant Physiol. 2001;126:811–25 11402209 10.1104/pp.126.2.811 PMC111171

[67] Lin H , Sun J, Hu Z. et al. Variation in glucosinolate accumulation among different sprout and seedling stages of broccoli (*Brassica oleracea* var. *italica*). Plants (Basel). 2022;11:1563 35736714 10.3390/plants11121563 PMC9227298

[68] Han N , Kim I, Kim J. et al. Tissue-specific distribution of primary and secondary metabolites of Baemoochae (x*Brassicoraphanus*) and its changes as a function of developmental stages. Food Res Int. 2021;150:110796 34865811 10.1016/j.foodres.2021.110796

[69] Xu D , Hunziker P, Koroleva O. et al. GTR-mediated radial import directs accumulation of defensive glucosinolates to sulfur-rich cells in the phloem cap of *Arabidopsis* inflorescence stem. Mol Plant. 2019;12:1474–84 31260813 10.1016/j.molp.2019.06.008

[70] Andersen TG , Nour-Eldin HH, Fuller VL. et al. Integration of biosynthesis and long-distance transport establish organ-specific glucosinolate profiles in vegetative *Arabidopsis*. Plant Cell. 2013;25:3133–45 23995084 10.1105/tpc.113.110890 PMC3784604

[71] Li B , Gaudinier A, Tang M. et al. Promoter-based integration in plant defense regulation. Plant Physiol. 2014;166:1803–20 25352272 10.1104/pp.114.248716 PMC4256871

[72] Sonderby IE , Hansen BG, Bjarnholt N. et al. A systems biology approach identifies a R2R3 MYB gene subfamily with distinct and overlapping functions in regulation of aliphatic glucosinolates. PLoS One. 2007;2:e1322 18094747 10.1371/journal.pone.0001322 PMC2147653

[73] Schweizer F , Fernandez-Calvo P, Zander M. et al. *Arabidopsis* basic helix-loop-helix transcription factors MYC2, MYC3, and MYC4 regulate glucosinolate biosynthesis, insect performance, and feeding behavior. Plant Cell. 2013;25:3117–32 23943862 10.1105/tpc.113.115139 PMC3784603

[74] Frerigmann H , Berger B, Gigolashvili T. bHLH05 is an interaction partner of MYB51 and a novel regulator of glucosinolate biosynthesis in *Arabidopsis*. Plant Physiol. 2014;166:349–69 25049362 10.1104/pp.114.240887 PMC4149720

[75] Hickman R , Van Verk MC, Van Dijken AJH. et al. Architecture and dynamics of the jasmonic acid gene regulatory network. Plant Cell. 2017;29:2086–105 28827376 10.1105/tpc.16.00958 PMC5635973

[76] Van Moerkercke A , Duncan O, Zander M. et al. A MYC2/MYC3/MYC4-dependent transcription factor network regulates water spray-responsive gene expression and jasmonate levels. Proc Natl Acad Sci USA. 2019;116:23345–56 31662474 10.1073/pnas.1911758116 PMC6859355

[77] Timm S , Florian A, Jahnke K. et al. The hydroxypyruvate-reducing system in *Arabidopsis*: multiple enzymes for the same end. Plant Physiol. 2011;155:694–705 21205613 10.1104/pp.110.166538 PMC3032460

[78] Kingsford C . A fast, lock-free approach for efficient parallel counting of occurrences of k-mers. Bioinformatics. 2011;27:764–70 21217122 10.1093/bioinformatics/btr011 PMC3051319

[79] Ranallo-Benavidez TR , Jaron KS, Schatz MC. GenomeScope 2.0 and Smudgeplot for reference-free profiling of polyploid genomes. Nat Commun. 2020;11:1432 32188846 10.1038/s41467-020-14998-3 PMC7080791

[80] Wang J , Mao S, Liang M. et al. Preharvest methyl jasmonate treatment increased glucosinolate biosynthesis, sulforaphane accumulation, and antioxidant activity of broccoli. Antioxidants. 2022;11:1298 35883789 10.3390/antiox11071298 PMC9312100

